# The clinicopathological analysis of ocular and orbit tumors in southeast of China

**DOI:** 10.3389/fonc.2023.1118862

**Published:** 2023-06-19

**Authors:** Yuan Lin, Xiaodong Liu, Yujie Zhang, Zhiwen Xie, Xie Fang, Ke Shi, Yanlin Zhong, Shengqi Su, Minqing Cai, Huping Wu, Shangkun Ou

**Affiliations:** ^1^ Eye Institute of Xiamen University and affiliated Xiamen Eye Center, School of Medicine, Xiamen University, Xiamen, Fujian, China; ^2^ Fujian Provincial Key Laboratory of Corneal & Ocular Surface Diseases, Xiamen, Fujian, China; ^3^ Xiamen Science and Technology Middle School, Xiamen, Fujian, China

**Keywords:** ocular tumor, benign tumor, malignant tumor, clinicopathologic characteristics, Southeast of China

## Abstract

**Purpose:**

The purpose of this study is to describe the clinicopathologic characteristics of ocular surface and orbit tumors in the Southeast of China and explore the method to differentiate the benign and malignant masses.

**Materials and methods:**

3468 patients undergoing mass resection from January 2015 to December 2020 were selected as observation subjects and were classified into benign and malignant masses according to postoperative pathology. The clinicopathologic characteristics were collected, including gender, age, pathological tissue signs, and pathological signs. Multivariate Logistic regression analysis of independent risk factors of malignant mass was applied to establish a diagnostic model and the efficacy was evaluated by the subject working characteristics (ROC) curve.

**Results:**

Benign tumors accounted for 91.5% of all cases, and malignant tumors accounted for 8.5%. The most common ocular benign tumors were nevi (24.2%), granuloma (17.1%), and cysts (16.4%). The most common ocular malignant tumors were malignant lymphoma (32.1%) and Basal cell carcinoma (20.2%). As for the histologic origin, melanocytic origin was on the list with 819 (23.6%), mesenchymal 661 (19.1%), epithelial 568 (16.3%), cystic 521 (15.0%), skin adnexal 110 (3.1%), lymphoid 94 (2.8%), and Neural 25(0.8%). Based on the gender, age, tumor location, and the pathological tissue image feature (including differentiation, structural atypia, covering epithelial, keratosis, nest structure/distribution, nuclear atypia, cytoplasmic change and nuclear division), the diagnostic model had predictive value to differentiate the benign and malignant masses.

**Conclusion:**

Most ocular surface and orbit tumors are benign. Tumor diagnosis is relative to the patient’s age, gender, tumor location, and pathologic characteristics. We generated a satisfactory diagnostic model to differential diagnosis of benign and malignant masses.

## Introduction

The ocular surface and orbit are a delicate and complex system including cornea and its adjacent protective structures: the limbus, the conjunctiva, the eyelids and their meibomian glands, the lacrimal glands, and the nerves (1). The ocular surface and orbit are exposed to the surrounding environment and breaks complex environmental balance which may eventually develop into eye tumors. The incidence, clinical characteristics, and disease severity of ocular surface and orbit tumors vary across regions and ethnicity (2, 3). Owing to the similar clinical manifestations, some ocular surface and orbit lesions are relatively complex for diagnosis (4, 5). For malignant tumors, it is difficult to distinguish in clinical due to overlapping features such as an orbital dilated mass or exophthalmos (5). While any misdiagnosis or missed diagnosis of malignant ocular tumors may compromise the patient’s vision and life. For example, conjunctival melanoma has a high degree of recurrence and metastasis that the total mortality rate can be as high as 25% (6). Several published studies (7–9) have provided information on the incidence of ocular surface lesions from a pathological perspective.

In this study, we retrospectively analyzed the histopathology and the onset of a sizeable pathological sample of 3690 eyes of 3468 patients with local resection of ocular surface and orbit tumor specimens admitted to the Xiamen Eye Centre of Xiamen University in southeast of China from 2015 to 2020. The aim of this study is to establish a prediction model to distinguish benign and malignant ocular masses and provide an evaluation basis for clinical diagnosis.

## Materials and methods

### The concept of the benign and malignant tumors

The diagnostic criteria, pathological features of tumors were based on WHO Classification of Tumors of the Eye (10). The tumors are classified depending on the size, tumor location, and extent of involvement. We classified histopathologic data that could not be defined by current systems as “other” in our study. Benign tumors refer to tumors that remain in the primary site without invading other parts of the body and will not spread to local structures or distant parts of the body that often grow slowly and have clear boundaries. The rapid mass growth, color, increased blood vessels, surface ulceration, bleeding, and other signs should be highly suspected of malignant changes that were overgrowing, easy to rupture, bleeding, and have unclear borders and odor or foul odor (11).

### Ethical approval

The clinical records of all patients with an ocular surface and orbit tumor, examined and treated by the Department of the ocular surface at Xiamen eye center between January 2015 and December 2020 were reviewed in the present study. Ethical approval for this study was obtained from Ethics Committee of Xiamen University and all the procedures were in accordance with the declaration of Helsinki. Informed consent was waived due to the retrospective nature of the study. The age and gender data of patients were collected at the initial examination. The clinical data included eye laterality, general anatomic location (involving the orbit, eyelid, conjunctiva, corneal, corneal limbus), general diagnostic category, and tumor diagnosis. The pterygium was excluded.

### Hematoxylin and eosin Staining

Surgically resected tissue specimens were fixed with 4% paraformaldehyde immediately followed by embedding in OCT for histological analysis. Sections of tissue were stained with hematoxylin and eosin (H&E) following the manufacturer’s protocol. The histopathological characteristics, including differentiation, structural atypia, inflammatory cell infiltration, hyperplasia, covering epithelium, keratinize, nested distribution/structure, nucleus pleomorphism, cytoplasmic changes, nuclear division, and histologic origin were analyzed referring to the published researches. The H&E staining results of all sections were reviewed and evaluated independently by two experienced pathologists from the Pathology of our hospital.

### Statistical analysis

Statistical analysis was performed using SPSS 25.0 Statistical Software (SPSS Inc., Chicago 2017, USA). Continuous variables were represented as the mean± standard deviation (SD). The Chi-square test was used for analysis. A *P-value*<0.05 was considered statistically significant. Multivariate Logistic regression analysis was used, and demographic characteristics, pathological tissue image performance, and combined predictors were analyzed by the receiver working characteristic (ROC) curve. α<0.05 was the inspection level.

## Results

The mean age of all 3468 patients was 39.4 ± 22.1year old (**Table 1**), ranging from newborn to 92-year-old. The number of patients with benign tumors was 3176(91.5%) with a mean age of 36.2 ± 21.3-year-old, and 292(8.5%) were malignant with a mean age of 57.3 ± 15.5-year-old (**Table 1**). Of the patients, the male-to-female ratio was 0.71:1 (1322/1853) for benign tumors and 1.35:1 (168/124) for malignant tumors. We found the Age and sex (X^2 =^ 27.573, P<0.001) of all patients were associated with different lesions.

**Table 1:** Characteristics of the study population.

Classification	Number	Mean (range) age, years	Gender				
			Male	Female	Sex ratio	X^2^	P value
Benign tumors	3176 (91.5%)	36.2 ± 21.3	1322 (86.3%)	1853 (86.7%)	0.71:1	27.573	<0.001
Malignant tumors	292 (8.5%)	57.3 ± 15.5	168 (13.7%)	124 (13.3%)	1.35:1		
Total	3468 (100%)	39.4 ± 22.1	1532 (100%)	2136 (100%)	0.72:1		

All cases were divided into five anatomical categories: orbit, eyelid, conjunctiva, corneal, and corneal limbus (**Table 2**). Regarding the 3176 cases of benign tumors, the eyelid was the most frequently occurring site (n=1703, 53.6%), followed by the conjunctiva (n=1093, 34.4%), orbit (n=201, 6.3%), corneal limbus (n=113, 3.5%), and corneal (n=66, 2.0%). Of the malignant tumors, 131 (44.9%) were eyelid tumors, 49 (16.9%) conjunctiva tumors, 73 (25%) orbit tumors, 28 (9.5%) corneal tumors, and 11 (3.7%) corneal limbus tumors. Regarding the occurring eyes, there were 1443 (45.4%) benign masses on the right, 1516 (47.7%) on the left, and 217 (6.9%) in bilateral eyes. Of the malignant tumors, 134 (45.8%) in the right, 153 (52.3%) on the left, and 5 (1.9%) in bilateral eyes.

**Table 2:** Anatomical classification of benign and malignant ocular tumors.

Classification	Tumor Location					Eye Site		
	Orbit	Eyelids	Conjunctiva	Cornea^#^	Corneal limbus	Right	Left	Bilateral
Benign tumors (n=3176)	201 (73.3%)	1703 (92.9%)	1093 (95.8%)	66 (70.2%)	113 (91.1%)	1443 (91.5%)	1516 (90.1%)	217 (97.7%)
Malignant tumors (n=292)	73 (26.7%)	131(7.1%)	49 (4.2%)	28 (29.8%)	11 (8.9%)	134 (8.5%)	153 (9.9%)	5 (2.3%)
Total (n=3468)	274 (100%)	1834 (100%)	1142 (100%)	94 (100%)	124 (100%)	1577 (100%)	1669(100%)	222 (100%)

^#^The cornea was excepted the tumor on the limbus.

The frequency of clinicopathological subtypes and demographic statistics (age, sex ratio) of patients were shown at **Tables 3**, **4** respectively. The prevalence of diverse types benign ocular surface tumors is different: nevus (n=771, 24.2%), granuloma (n=542, 17.1%) and cyst (n=521, 16.4%), which form almost two-thirds of total ocular surface tumors (57.7%). The highest constituent ratio of malignant tumors was Malignant lymphoma (n=94, 32.1%), followed by Basal cell carcinoma (n=59, 20.2%).

**Table 3:** Characteristics of benign tumors patients.

Benign	Number	Mean (range) age, years	Gender				
			Male	Female	Sex ratio	X^2^	P value
Nevus	771 (24.2%)	34.5 ± 17.2	235	536	0.43:1	32.331	<0.001
Granuloma	542 (17.1%)	36.4 ± 22.7	254	288	0.88:1	5.177	0.023
Cyst	521 (16.4%)	37.6 ± 23.5	253	268	0.94:1	8.771	0.003
Lipoma	239 (7.5%)	39.8 ± 23.8	135	104	1.29:1	20.029	<0.001
Squamous papilloma	181 (5.6%)	43.5 ± 19.6	100	81	1.23:1	12.991	<0.001
Dermoid tumor	167 (5.2%)	14.7 ± 17.1	92	75	1.22:1	11.762	0.001
Xanthoma	161 (5.0%)	46.5 ± 9.4	31	130	0.23:1	31.843	<0.001
Hemangioma	142 (4.5%)	37.4 ± 19.8	53	89	0.59:1	1.042	0.307
Seborrheic keratosis	94 (2.9%)	54.5 ± 15.2	32	62	0.51:1	2.171	0.141
Fibroma	93 (2.9%)	36.1 ± 18.9	41	52	0.78:1	0.223	0.637
Chalazion	56 (1.7%)	18.8 ± 20.1	22	34	0.64:1	0.125	0.723
Melanosis	35 (1.2%)	28.2 ± 10.3	7	28	0.25:1	6.681	0.01
Calcifying epithelioma	33 (1.0%)	13.8 ± 13.6	11	22	0.875:1	0.927	0.336
Adenoma	24 (0.8%)	43.1 ± 18.2	8	16	0.5:1	0.676	0.411
Denaturation	19 (0.6%)	48.9 ± 13.7	9	10	0.9:1	0.255	0.613
Hyperplasia	15 (0.5%)	39.8 ± 24.8	7	8	0.87:1	0.155	0.693
Verruca vulgaris	13 (0.5%)	45.1 ± 24.4	4	9	0.44:1	0.630	0.428
Nerve sheath tumor	11 (0.5%)	33.2 ± 17.1	4	7	0.57:1	0.125	0.723
Osteoma	5 (0.2%)	30.8 ± 12.1	1	4	0.25:1	0.962	0.327
Schwannoma	4 (0.1%)	24.0 ± 5.2	1	3	0.33:1	0.455	0.500
Pleomorphic adenoma	3 (0.1%)	56.0 ± 10.1	2	1	2:1	0.773	0.379
Others	46 (1.5%)	46.8 ± 19.2	20	26	0.68:1	0.013	0.908
Total	3176 (100%)	36.2 ± 21.3	1322	1853	0.71:1		

**Table 4:** Characteristics of malignant tumors patients.

Malignant	Number	Mean (range) age, years	Gender				
			Male	Female	Sex ratio	X^2^	P value
Lymphoma	94 (32.1%)	54.0 ± 13.2	69	25	2.76:1	7.556	0.006
Basal cell carcinoma	59 (20.2)	54.0 ± 13.2	29	30	0.96:1	1.400	0.237
Sebaceous adenocarcinoma	35 (11.9%)	61.8 ± 12.2	14	21	0.66:1	3.893	0.048
Malignant epithelial Hyperplasia	34 (11.7%)	50.0 ± 20.0	17	17	1:01	0.704	0.401
Squamous cell Carcinoma	18 (6.2%)	62.7 ± 12.7	12	6	2:01	0.581	0.446
Malignant Melanoma	13 (4.4%)	62.8 ± 13.2	6	7	0.85:1	0.658	0.417
inflammatory Myofibroblastic tumor	9 (3.1%)	33.2 ± 10.5	3	6	0.5:1	2.084	0.149
Preinvasive Carcinoma	7 (2.4)	59.8 ± 14.5	5	2	2.5:1	0.541	0.462
Epithelioma	5 (1.8%)	65.2 ± 6.4	5	0	5/0	3.645	0.056
Sebaceous gland carcinoma	1 (<0.1%)	63.0 ± 0	0	1	0/1	1.349	0.246
Fibromyxosarcoma	1 (<0.1%)	49.0 ± 0	0	1	0/1	1.349	0.246
Teratoma	1 (<0.1%)	3.0 ± 0	0	1	0/1	1.349	0.246
Others	16 (6.1%)	51.5 ± 22.7	7	9	0.77:1	1.184	0.277
Total	292 (100%)	57.3 ± 15.5	168	124	1.35:1		

Among benign tumors, nevus (n=497, 15.6%), cysts (n=279, 8.7%), granuloma (n=276, 8.6%), xanthoma (n=161, 4.9%), squamous papilloma (n=95, 2.9%), and seborrheic keratosis (n=93, 2.9%) were the most frequent and accounted together for 43.6% of all eyelid cases (**Table 5**). Among the malignant tumors, the eyelids (n=131, 44.9%), conjunctiva (n=49, 16.9%), and orbit (n=73, 25.0%) were the most frequent and accounted together for 86.8% of all malignant tumor cases (**Table 6**).

**Table 5:** Anatomical classification of benign ocular tumors.

Benign	Tumor Location						Eye site	
	Orbit	Eyelids	Conjunctiva	Corneal	Corneal limbus	Total	Laterality	Bilateral
Nevus	1 (0.5%)	497 (29.2%)	266 (24.4%)	3 (4.5%)	4 (3.5%)	771 (24.3%)	763 (25.8%)	8 (3.7%)
Granuloma	11 (5.5%)	276 (16.2%)	236 (21.6%)	10 (15.2%)	9 (7.9%)	542 (17.1%)	511 (17.3%)	31 (14.3%)
Cyst	32 (15.9%)	279 (16.4%)	210 (19.2%)	0 (0%)	0 (0%)	521 (16.4%)	516 (17.4%)	5 (2.3%)
Lipoma	32 (15.9%)	43 (2.5%)	159 (14.5%)	2 (3.0%)	3 (2.7%)	239 (7.5%)	201 (6.8%)	38 (17.5%)
Squamous papilloma	0 (0%)	95 (5.6%)	79 (7.2%)	2 (3.0%)	5 (4.4%)	181 (5.7%)	178 (6.0%)	3 (1.4%)
Dermoid tumor	2 (0.9%)	18 (1.1%)	30 (2.7%)	32 (48.5%)	85 (75.2%)	167 (5.2%)	166 (5.6%)	1 (0.5%)
Xanthoma	2 (0.9%)	157 (9.2%)	2 (0.2%)	0 (0%)	0 (0%)	161 (5.1%)	51 (1.7%)	110 (50.6%)
Hemangioma	71 (35.5%)	54 (3.2%)	16 (1.5%)	0 (0%)	1 (0.9%)	142 (4.5%)	142 (4.8%)	0 (0%)
Seborreic keratosis	1 (0.5%)	93 (5.5%)	0 (0%)	0 (0%)	0 (0%)	94 (2.9%)	89 (3.0%)	5 (2.3%)
Fibroma	11 (5.5%)	51 (3.1%)	26 (2.4%)	2 (3.0%)	3 (2.7%)	93 (2.9%)	92 (3.1%)	1 (0.5%)
Chalazion	1 (0.5%)	51 (3.1%)	4 (0.4%)	0 (0%)	0 (0%)	56 (1.8%)	44 (1.5%)	12 (5.5%)
Melanosis	0 (0%)	0 (0%)	34 (3.2%)	0 (0%)	1 (0.9%)	35 (1.1%)	35 (1.2%)	0 (0%)
Adenoma	6 (2.9%)	8 (0.5%)	7 (0.6%)	0 (0%)	0 (0%)	21 (0.7%)	21 (0.7%)	0 (0%)
Denaturation	0 (0%)	1 (<0.1%)	15 (1.4%)	3 (4.6%)	0 (0%)	19 (0.6%)	17 (0.6%)	2 (0.9%)
Hyperplasia	1 (0.5%)	1 (<0.1%)	3 (0.3%)	9 (13.6%)	1 (0.9%)	15 (0.5%)	15 (0.5%)	0 (0%)
Calcifying epithelioma	1 (0.5%)	31 (1.8%)	1 (<0.1%)	0 (0%)	0 (0%)	33 (1.1%)	33 (1.1%)	0 (0%)
Nerve sheath tumor	13 (6.5%)	2 (0.1%)	0 (0%)	0 (0%)	0 (0%)	15 (0.5%)	15 (0.5%)	0 (0%)
Verruca vulgaris	0 (0%)	13 (0.7%)	0 (0%)	0 (0%)	0 (0%)	13 (0.4%)	13 (0.4%)	0 (0%)
Osteoma	3 (1.5%)	1 (<0.1%)	1 (<0.1%)	0 (0%)	0 (0%)	5 (0.1%)	5 (0.2%)	0 (0%)
Schwannoma	3 (1.5)	1 (<0.1%)	0 (0%)	0 (0%)	0 (0%)	4 (0.1%)	4 (0.1%)	0 (0%)
Pleomorphic adenoma	2 (0.9%)	1 (<0.1%)	0 (0%)	0 (0%)	0 (0%)	3 (0.1%)	3 (0.1%)	0 (0%)
Others	8 (4.1%)	30 (1.7%)	4 (0.4%)	3 (4.6%)	1 (0.9%)	46 (1.4%)	45 (1.6%)	1 (0.5%)
Total	201 (100%)	1703 (100%)	1093 (100%)	66 (100%)	113 (100%)	3176 (100%)	2959 (100%)	217 (100%)

**Table 6:** Anatomical classification of common malignant ocular tumors.

Malignant	Tumor Location						Eye site	
	Orbit	Eyelids	Conjunctiva	Corneal	Corneal limbus	Total	Laterality	Bilateral
Lymphoma	58 (79.4%)	11 (8.3%)	25 (51.1%)	0 (0%)	0 (0%)	94 (32.2%)	41 (30.6%)	5 (100%)
Basal cell carcinoma	0 (0%)	57 (43.5%)	1 (2.1%)	1 (3.6%)	0 (0%)	59 (20.2%)	29 (21.6%)	0 (0%)
Sebaceous adenocarcinoma	0 (0%)	33 (25.2%)	2 (4.0%)	0 (0%)	0 (0%)	35 (11.9%)	20 (14.9%)	0 (0%)
Malignant epitheliod mesothelioma	0 (0%)	5 (3.8%)	9 (18.3%)	16 (57.1%)	4 (36.4%)	34 (11.6%)	14 (10.5%)	0 (0%)
Squamous cell carcinoma	0 (0%)	6 (4.6%)	5 (10.1%)	4 (14.3%)	3 (27.2%)	18 (6.2%)	6 (4.5%)	0 (0%)
Malignant melanoma	2 (2.7%)	7 (5.4%)	3 (6.1%)	0 (0%)	1 (9.2%)	13 (4.5%)	6 (4.5%)	0 (0%)
inflammatory myofibroblastic tumor	6 (8.3%)	2 (1.5%)	1 (2.1%)	0 (0%)	0 (0%)	9 (3.1%)	5 (3.7%)	0 (0%)
preinvasive carcinoma	0 (0%)	1 (0.7%)	2 (4.1%)	4 (14.3%)	0 (0%)	7 (2.4%)	2 (1.5%)	0 (0%)
Epithelioma	0 (0%)	0 (0%)	0 (0%)	2 (7.1%)	3 (27.2%)	5 (1.8%)	3 (2.2%)	0 (0%)
Sebaceous gland carcinoma	0 (0%)	1 (0.7%)	0 (0%)	0 (0%)	0 (0%)	1 (0.3%)	0 (0%)	0 (0%)
fibromyxosarcoma	1 (1.4%)	0 (0%)	0 (0%)	0 (0%)	0 (0%)	1 (0.3%)	0 (0%)	0 (0%)
Teratoma	1 (1.4%)	0 (0%)	0 (0%)	0 (0%)	0 (0%)	1 (0.3%)	1 (0.7%)	0 (0%)
Others	5 (6.8%)	8 (6.3%)	1 (2.1%)	1 (3.6%)	0 (0%)	15 (5.2%)	7 (5.3%)	0 (0%)
Total	73 (100%)	131 (100%)	49 (100%)	28 (100%)	11 (100%)	292 (100%)	134 (100%)	5 (100%)

Furthermore, we analyzed the subtypes of histologic origins of ocular mass lesions. Among all the 3468 cases, melanocytic origin was on the list with 819 (23.6%), mesenchymal 661 (19.1%), epithelial 568 (16.3%), cystic 521 (15.0%), skin adnexal 110 (3.1%), lymphoid 94 (2.8%), Neural 25(0.8%) and others 670 (19.3%) (**Table 7**).

**Table 7:** The histologic origin of benign and malignant tumors and tumor-like lesions.

Histologic origin	Benign	Malignant	Benign/Malignant	X^2^	P value	Total
Cystic	521 (16.4%)	0 (0%)	521/0	47.332	<0.001	521 (15.0%)
Melanocytic	806 (25.4%)	13 (4.4%)	1:0.01	46.804	<0.001	819 (23.6%)
Epithelial	448 (14.2%)	120 (41.2%)	1:0.26	85.975	<0.001	568 (16.3%)
Skin adnexal	72 (2.2%)	38 (13.2%)	1:0.52	86.919	<0.001	110 (3.1%)
Mesenchymal	652 (20.6%)	9 (3.1%)	1:0.01	40.924	<0.001	661 (19.1%)
Neural	15 (0.4%)	10 (3.1%)	1:0.67	31.341	<0.001	25 (0.8%)
Lymphoid	0 (0%)	94 (32.2%)	0/94	794.394	<0.001	94 (2.8%)
Others	662 (20.8%)	8 (2.8%)	1:0.01	43.600	<0.001	670 (19.3%)
Total	3176 (100%)	292 (100%)				3468 (100%)

**Figure 1:**
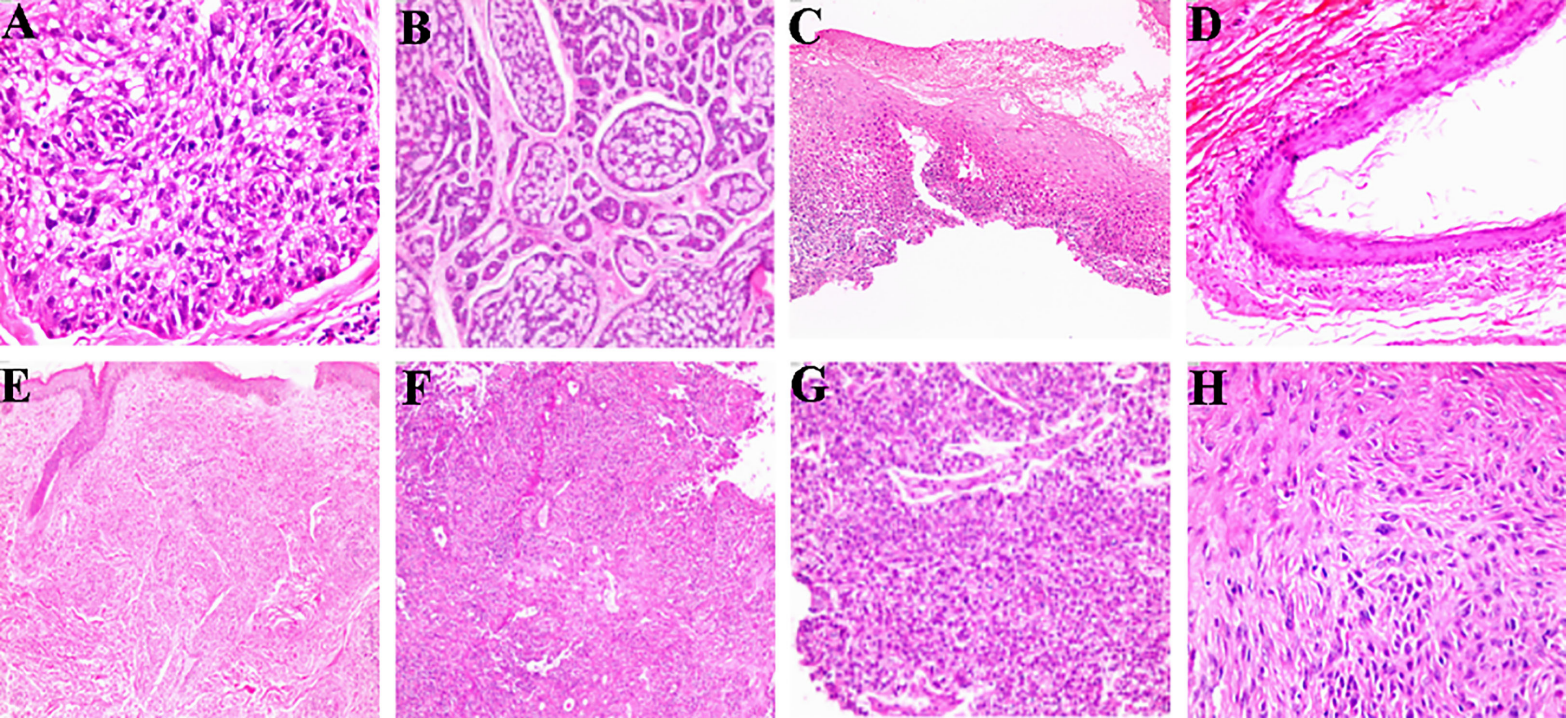
The pathological tissue image feature of ocular benign and malignant tumor. **(A)** Differentiation (HE×200); **(B)** Structural atypia (HE×100); **(C)** Covering epithelium (HE×40); **(D)** Keratosis (HE×100); **(E)** Nest structure/distribution (HE×100); **(F)** nuclear atypia (HE×100); **(G)** Cytoplasmic change (HE×100); **(H)** nuclear division (HE×200).

**Figure 2:**
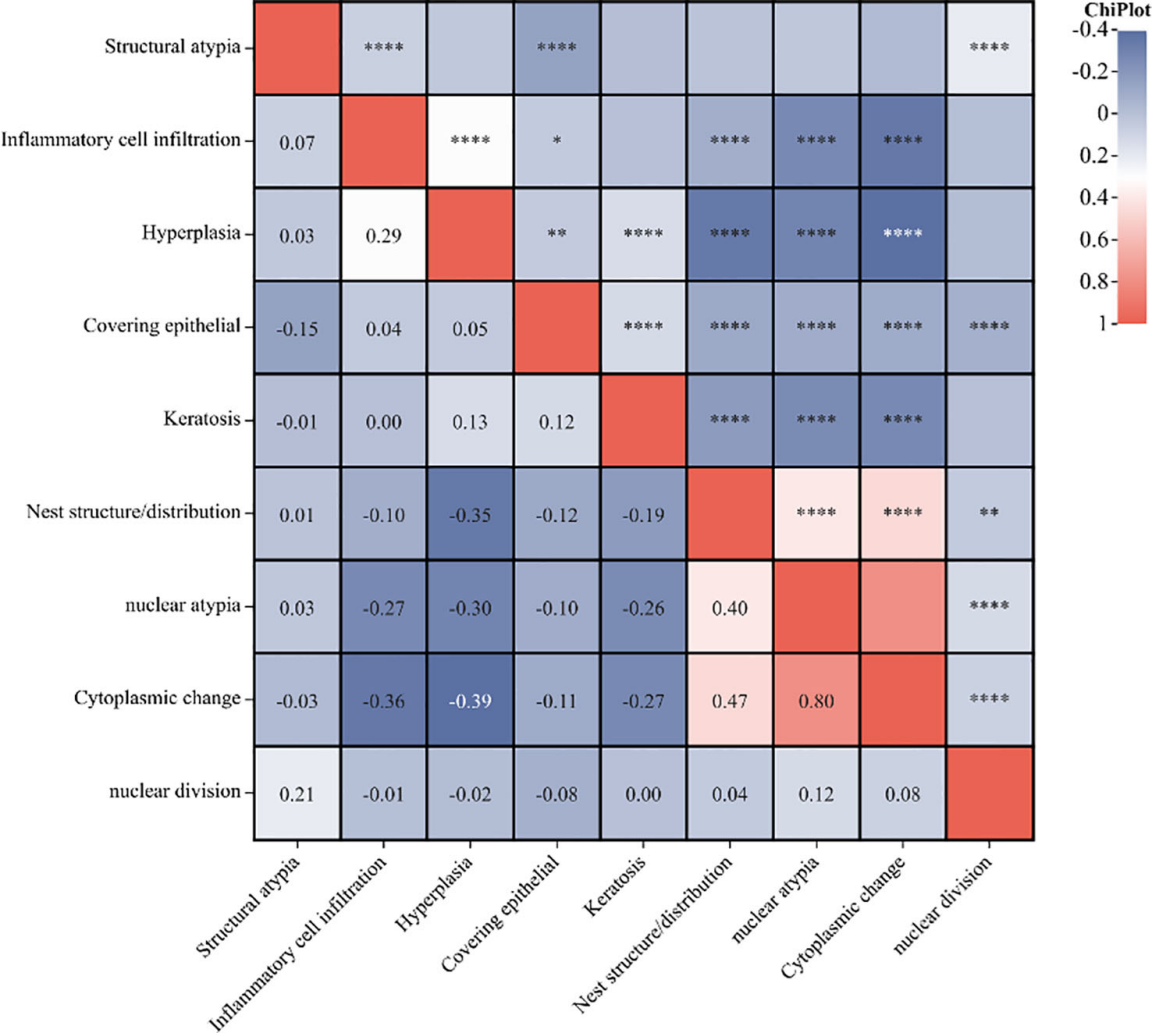
The correlation heatmap of the histopathological characteristics. (*P<0.05, **P<0.01, ****P<0.0001).

**Figure 3:**
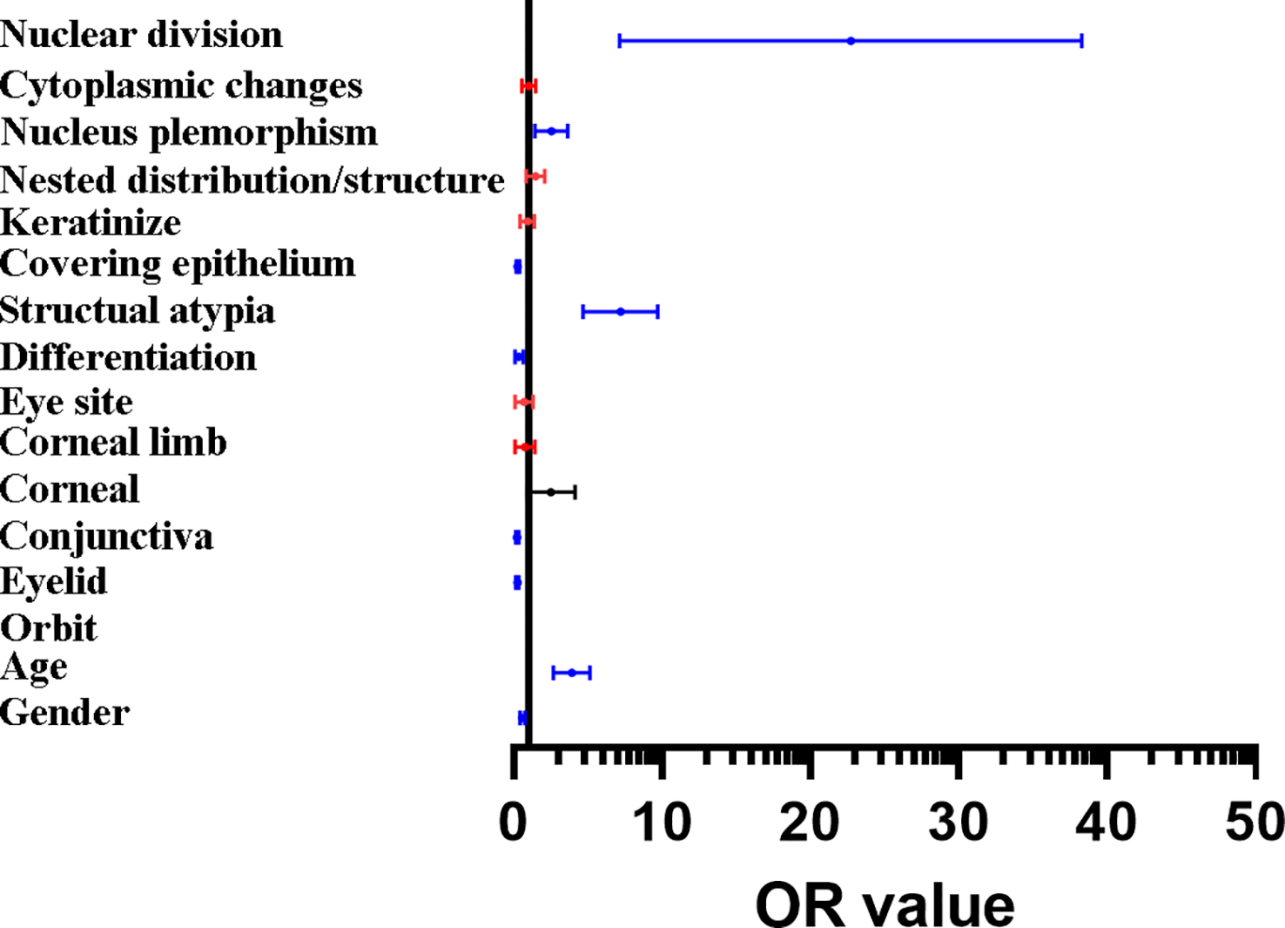
Forest map of the results of binary logistic regression. The histopathological features include nuclear, nucleus plemorphism, covering epithelium, structual atypia, differentiation, the location of conjunctiva and eyelid, in addition to age and gender could be the independent diagnostic factors of the malignant tumors and tumor-like lesions. Red line: the variables with no independent predictive power (p > 0.05), blue line: the variables are showing significant independent predictive power (p < 0.01), black line: the variables are showing a specific independent predictive power (0.05 > p > 0.01).

**Figure 4:**
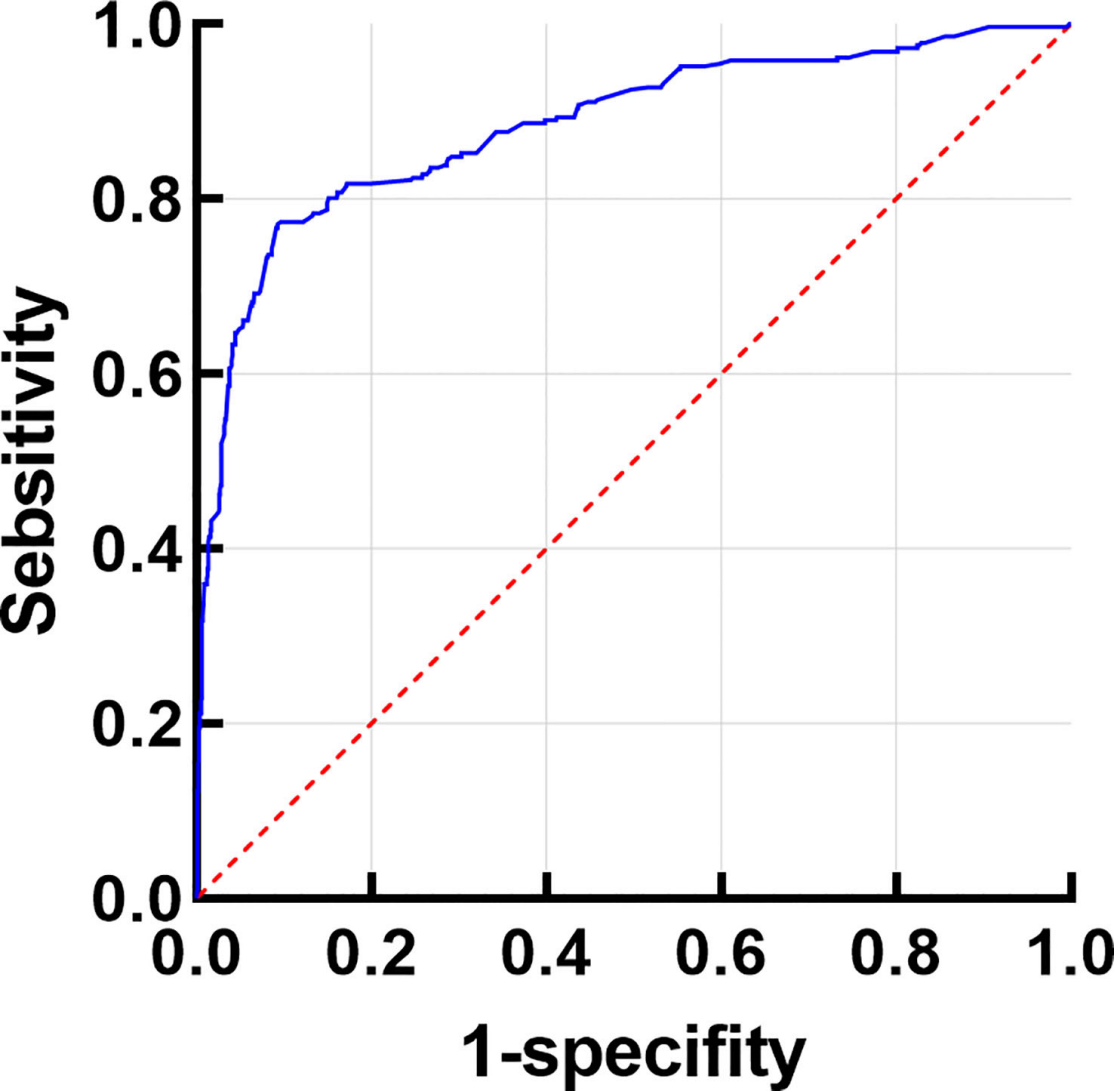
Receiver operating characteristic (ROC) curve analysis. The ROC curve value was 0.884(0.860-0.907, P<0.001).

When classified and collected information of the different histopathological features of benign and malignant tumor lesions, we found there were some similar histopathological features between benign and malignant. We suggested it has the value to explore the correlation of histopathological image features to different the properties of ocular tumor lesions (**Figure 1**). According to the correlation analysis, some the histopathological feature could become the diagnostic factor to different the property of the ocular tumor (**Figure 2** and **Table 8**), such as Gender, age, tumor location, eye site, differentiation, structural atypia, covering epithelium, keratosis, nest structure/distribution, nuclear atypia, cytoplasm changes, and nuclear division (all P <0.05). Using pathological results as the dependent variable (0= ocular benign tumor, 1= ocular malignant tumor), a mathematical model was established to analyze the statistically significant variables above. The univariate statistical analysis was set as follows: gender (male =0. Female =1), age (≦60 = 0, > 60 = 1), tumor location (orbit =0, eyelid =1, Conjunctive =2, Corneal =3, Corneal limbus =4), eye site(laterality =0, bilateral=1), the pathological tissue image performance includes differentiation (No=0, Yes=1), structural atypia (Discipline=0, Disorderly=1), covering epithelial (No=0, Yes=1), keratosis (No=0, Yes=1), nest structure/distribution (No=0, Yes=1), nuclear atypia (No=0, Yes=1), cytoplasmic change (No=0, Yes=1), nuclear division (No=0, Yes=1) (**Table 8**). The logistic regression model was established as an independent variable (X), using the stepwise method (Forward: LR method). The results showed that sex, age, tumor location, differentiation, structural atypia, covered epithelium, nuclear atypia, and nuclear division were the independent influencing factors in the diagnostic and differential diagnosis of benign and malignant ocular masses (**Table 9** and **Figure 3**). We used the ROC curve to test the model of the all-independent diagnostic factors according to the multivariate Logistic regression. the largest area under the ROC curve value was 0.884(0.860-0.907, P<0.001). It showed that was a reliable diagnostic model to different the benign and malignant (**Figure 4**).

**Table 8:** General Data and clinicopathologic image in benign group and malignant group.

Characteristic	Benign group	Malignant group	χ2	P
**Gender**			27.573	<0.001
Male	1322	168		
Female	1853	124		
**Age (adjusted)**			173.152	<0.001
≤60	2726	163		
>60	450	129		
**Tumor Location**			196.885	<0.001
Orbit	201	73		
Eyelids	1703	100		
Conjunctiva	1093	80		
Corneal	66	28		
Corneal limbus	113	11		
**Eye Site**			11.701	0.001
Laterality	2959	287		
Bilateral	217	5		
**Differentiation**			35.218	<0.001
Yes	365	1		
No	2811	291		
**Structural atypia**			421.704	<0.001
Discipline	3021	180		
Disorderly	155	112		
**Inflammatory cell infiltration**			2.015	0.156
Yes	1009	81		
No	2167	211		
**Hyperplasia**			1.992	0.158
Yes	1407	121		
No	1669	171		
**Covering epithelium**			157.031	<0.001
Yes	1716	51		
No	1360	241		
**Keratinize**			7.848	0.005
Yes	513	29		
No	2663	263		
**Nested distribution/structure**			17.735	<0.001
Yes	590	84		
No	2586	208		
**Nucleus pleomorphism**			54.471	<0.001
Yes	1242	179		
No	1934	113		
**Cytoplasmic changes**			30.130	<0.001
Yes	1158	154		
No	2018	138		
**Nuclear division**			464.016	<0.001
Yes	14	55		
No	3162	237		

**Table 9:** Results of the univariate and multivariate Logistic regression of diagnostic factors related to malignant (full model, no. of patients =3468).

Variables	Univariate analysis			Multivariate analysis		
	OR	OR (95%CI)	P	OR	OR (95%CI)	P
Gender	0.526	0.413-0.671	<0.001	0.583	0.430- 0.791	0.001
Age	4.861	3.781-6.251	<0.001	3.783	2.746-5.211	<0.001
Orbit			<0.001			<0.001
Eyelid	0.206	0.150-0.284	<0.001	0.238	0.156-0.364	<0.001
Conjunctiva	0.120	0.081-0.177	<0.001	0.218	0.135-0.352	<0.001
Corneal	1.056	0.624-1.786	0.840	2.156	1.090-4.267	0.027
Corneal limbus	0.261	0.133-0.512	<0.001	0.576	0.221-1.504	0.26
Eye Site	0.238	0.097-0.581	0.002	0.540	0.210-1.392	0.202
Differentiation	0.189	0.089-0.404	<0.001	0.294	0.130-0.663	0.003
Structural atypia	12.304	9.248-16.370	<0.001	6.919	4.857-9.857	<0.001
Inflammatory cell infiltration	0.824	0.631-1.077	0.156			
Hyperplasia	0.890	0.698-1.135	0.346			
Covering epithelium	0.180	0.132-0.245	<0.001	0.275	0.187-0.405	<0.001
Keratinize	0.594	0.403-0.877	0.009	0.835	0.489-1.427	0.509
Nested distribution/structure	1.770	1.353-2.316	<0.001	1.383	0.902-2.122	0.137
Nucleus pleomorphism	3.036	2.358-3.908	<0.001	2.391	1.533-3.728	<0.001
Cytoplasmic changes	1.945	1.528-2.474	<0.001	0.952	0.599-1.514	0.836
Nuclear division	52.414	28.726-95.637	<0.001	19.159	9.231-39.763	<0.001

## Discussion

In this study, we have analyzed the clinicopathological characteristics with a large cohort (>3000 cases) of ocular and orbit tumors in Southeast of China. In addition, we provide, for the first time a predictive value method to differentiate the ocular surface and orbit benign and malignant masses.

The ocular surface contact with the outside environment directly, which is frequently invaded by various noxious pathogens. Clinical symptoms cannot be used to diagnose all ocular surface and orbit tumors accurately as many ocular surface and orbit masses have similar signs and features, while the histopathological examination is the gold standard for confirming the diagnosis. To better identify ocular surface and orbit masses, collection of cases, differentiation of pathological types and epidemiological changes are needed. Different regions, races, gender, age, and epidemiology can usually lead to the diverse occurrence and development progresses of ocular surface and orbit masses (12–17). In our study, over 90% the tumors were benign, while malignant tumors accounted for 8.5%. The benign tumors were preferred to the younger female, while most the malignant tumors were elder male. The most common ocular surface and orbit benign tumors were nevi, granuloma and cysts. The most common ocular malignant tumors were malignant lymphoma, basal cell carcinoma and sebaceous adenocarcinoma. As for the histologic origin, most the tumors were originated from melanocytic, mesenchymal, epithelial, cystic, skin adnexal, lymphoid, and Neural. The benign tumors were the most common in the eyelid site, followed by the conjunctiva, orbit, cornea, and corneal limbus. The orbit and cornea had more risk to occur malignant tumors.

Orbital tumors are complex and diverse; and early lesions are hidden. Typical clinical manifestations include visible masses are accompanied by exophthalmos and limited mobility. The tissues in orbit are derived from mesoderm, epidermal ectoderm, and neuroectoderm (18), with rich internal blood vessels and nerves. The unique development and differentiation of the orbital tissue and the abundant blood supply cause diverse masses in orbit including the benign tumors, including hemangioma, lipoma, cyst (**Table 5** and **Supplementary Figure 1**); in addition, malignant masses of lymphoma, myofibroblastic, malignant melanoma were also observed in our reports (**Table 6** and **Supplementary Figure 1**). The results of our study shows that the most common orbital tumor is hemangioma, which is consistent with previous literature reports (19).

The eyelid is located at the outermost part of the eye structure and directly contacts with the outside environment, which results in the most frequent tumor occurrence (9). The eyelid tumor is the most common part of the eye tumor. In our study, the occurrences of benign lesions were more frequent than malignant lesions. In addition to inflammatory lesions, the top common benign lesions were nevus, cyst, xanthoma and granuloma (**Table 5** and **Supplementary Figure 2**). In other studies, the number of inflammatory lesions in the eyelid was shown in different proportions, while that of the common benign lesions were roughly the similar (20). Basal cell carcinoma (BCC) is a common ocular surface malignancy that almost occurs in the eyelid. BCC is usually manifested as nodules with pearl margins, central ulceration, and telangiectasia vessels (**Supplementary Figure 2**). The risk factors of BCC include old age, sunshine, and immunosuppression, with a high recurrence rate (21). In our study, BCC occurs in the eyelids with no apparent difference between men and women. BCC grew slowly and infiltrated locally but did not metastasize. Including the BCC, sebaceous adenocarcinoma and lymphoma were also common malignant tumors in eyelid (**Supplementary Figure 2**).

Conjunctiva is a thin, soft, smooth, elastic, and translucent mucous membrane between the eyelid. It’s a mucous membrane that consists of fibroblast, keratinized squamous epithelium, goblet cells, abundant blood and lymphatic vessels. Thus, conjunctival nerves, granuloma and cysts were common in conjunctiva (**Supplementary Figure 3**). Most of the malignant conjunctival tumors in our group are lymphoma, malignant epithelioid mesothelioma and Squamous cell carcinoma (**Supplementary Figure 3**). The organized lymphoid tissue in the conjunctiva have the chance over clonal proliferation to malignancy originating lymphoma (22); and the conjunctiva lymphoma may be a manifestation of systemic lymphoma (23).

The cornea is an essential optical pathway, and its structural and functional integrity is closely related to normal refraction and visual function. The growth of corneal tumors may cause severe visual impairment or even blindness, while malignant corneal tumors are also prone to metastasis. Dermoid tumor, granuloma, hyperplasia were the common benign tumors in cornea (**Supplementary Figure 4**). Dermoid tumors are the most common benign tumor in the cornea that have the appearance of skin, clear boundaries, and the hair tissue; larger ones can often cause corneal astigmatism and decrease vision. Besides, we also found some malignant corneal masses, including preinvasive carcinoma, malignant epithelium and intraepithelial neoplasia (**Supplementary Figure 4**).

The corneal limbus is the anatomical boundary between the transparent cornea and the white sclera covering the rich vascular conjunctiva. The corneal epithelial stem cells also locate in the limbus that have the potential proliferation (24). The unique anatomical structure and location make the limbus have special tumor characteristic (25). In our study, we collected the corneal limbal masses cases of dermoid tumors, granuloma and squamous papilloma that were most common in benign classification. Besides, malignant masses were observed with malignant epithelial, squamous cell carcinoma, and epithelial carcinoma (**Supplementary Figure 5**). The early appearance of squamous papilloma and squamous cell carcinoma are similar (26). However, squamous papillomas occur well at any age with red color and soft quality. Squamous cell carcinoma *in situ* occurs more in older adults over 60 and is more typical in men. The later stage is the cauliflower shape, With abundant neovascularization. However, squamous carcinoma blood vessels are more generous and usually occur in the eyelid fissure area unless it has granulation (8).

The occurrence and development of tumors originate from the changes and progress of abnormal pathological tissues caused by abnormal growth regulation of normal tissues under the influence of environment, genetics, mutation, etc. Nowadays, pathology has been comprehensively developed into the field of molecular biology, and many abnormal expressions of genes, signal pathways and proteins that lead to the occurrence and development of tumors have been constantly excavated. However, although the staining and fluorescence amplification of different markers based on pathological tissue samples showed specificity in different tumor types and sub-classifications, they were not enough to be used as independent diagnostic criteria for different tumors. Tumors masses have different histopathological origins, and we found a statistically significant difference between benign and malignant histopathological origins (**Table 7**). As a result, we suggest some relationship between histopathological characteristics and the tumors’ different properties. We tried to classify the various features of the histopathological image and collect information in every case. The correlation analysis shows a correlation between different classification features (**Figure 2**).

Therefore, we had the hypothesis that it’s available to find the diagnostic factors that can differentiate benign and malignant tumors through the pathological manifestations of different ocular surface and orbit tumors. We used the logistic regression model to learn that: sex, age, tumor location, differentiation, structural atypia, covered epithelium, nuclear atypia, and nuclear division were the independent influencing factors in the diagnostic and differential diagnosis of benign and malignant ocular masses (**Table 9**). According to the ROC curve analysis, the sex, age, tumor location, and mass of the pathological tissue image performance had some predictive value. However, the predictive value alone was not high. This study tried to use the Logistic regression model to fit demographic characteristics, clinical factors, and mass pathological tissue characteristic generation joint predictors. The sensitivity and specificity of our final diagnosis model were 0.771 and 0.907, respectively. The results found that the Area Under Curve (AUC) for benign and malignant mass diagnosis is 0.884, significantly higher than the above factors. Indicators alone provide a new way for the differential diagnosis of benign and malignant clinical eye mass, were conducive to timely taking reasonable preventive measures to prevent misdiagnosis, and improve patients’ quality of life. As a result, the Logistic regression model fitting to demographic characteristics, clinical factors, and mass pathological tissue characteristics has reliable diagnostic value for the differential diagnosis of benign and malignant mass and can provide a reliable basis for clinical diagnosis and treatment.

There were some limitations of this study. Firstly, the database was collected retrospectively; the prospective study should be future perform. Secondly, the number of patients with malignancy is significantly less than those with benign tumors, so the ability to compare with the more common benign disease may not detect all clinical differences. Furthermore, the diagnostic model in this study may not necessarily apply to different regions and races, because this study samples that were collected from the single center. All in all, we hope to add more samples and indexes in future studies and find the more valuable auxiliary inspections to extend our model to achieve the purpose of precision medical treatment.

In summary, this study analyzed the clinicopathological characteristics of a large cohort ocular and orbit tumors lesions in Southeast of China. The epidemiological, clinical, and histopathologic features of the tumors should be deeper understanding to compared with the classification in order to better evaluate the ocular surface and orbit lesions. It’s difficult to differentiate diagnosis of some benign and malignant tumors in clinical work. Based on the clinicopathological characteristics of the benign and malignant tumors, we generate a satisfactory prediction model for this disease to differentiate diagnosis of benign and malignant masses. With the improvement of diagnosis, the prospects of better treatment strategies will be brilliant.

## Data availability statement

The original contributions presented in the study are included in the article/**Supplementary Material**. Further inquiries can be directed to the corresponding authors.

## Ethics statement

The studies involving human participants were reviewed and approved by Ethics Committee of Xiamen University. Written informed consent to participate in this study was provided by the participants’ legal guardian/next of kin. Written informed consent was obtained from the individual(s), and minor(s)’ legal guardian/next of kin, for the publication of any potentially identifiable images or data included in this article.

## Author contributions

SO and HW designed the experiments. YL, XL, ZX, FX, YjZ, KS and YlZ carried out experiments. YL, XL and SO analyzed the data and wrote the manuscript. MC and SS assisted revise the manuscript. All authors contributed to the article and approved the submitted version.

## Supplementary material

The Supplementary Material for this article can be found online at: https://www.frontiersin.org/articles/10.3389/fonc.2023.1118862/full#supplementary-material .
