# Supplementary material for: The clinicopathological analysis of ocular and orbit tumors in southeast of China
Source: Front Oncol. 2023 Jun 19;13:1118862. doi: 10.3389/fonc.2023.1118862 (PMC10316389; doi:10.3389/fonc.2023.1118862)
Supplement: Supplementary file 1 [file DataSheet_1.docx]

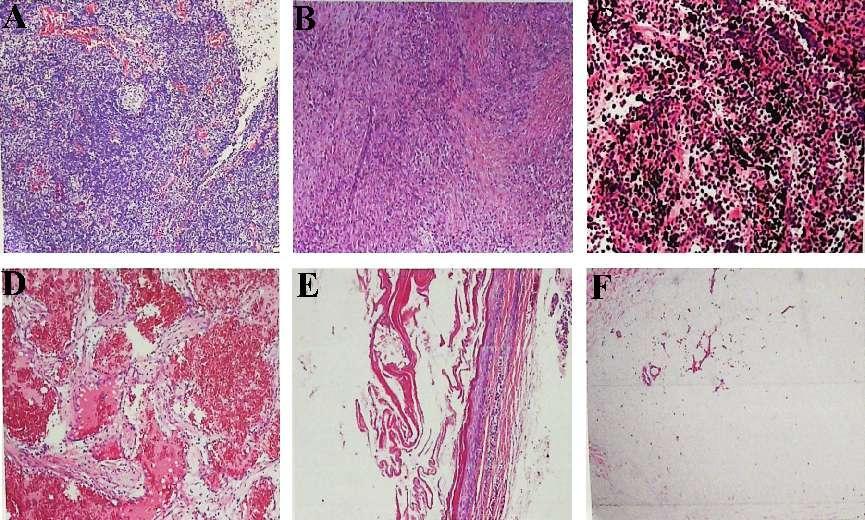
**S-Figure 1. The common masses of the orbit tumor.** (**A**) Lymphoma (HE×100); (**B**): Inflammatory myofibroblastic tumor (HE×100); (C) Malignant melanoma (HE×200); (**D**) Hemangioma (HE×100); (**E**) Cyst (HE×100); (**F**) lipoma (HE×100).


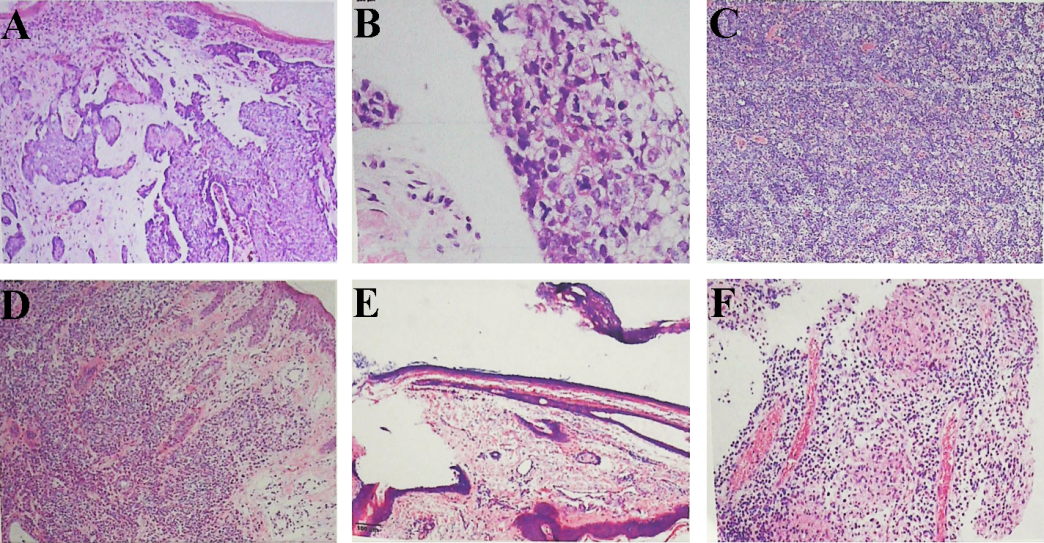
**S-Figure 2. The common masses of the eyelid tumor.** (**A**) Basal cell carcinoma (HE×100); (**B**)Sebaceous adenocarcinoma (HE×200); (**C**) Lymphoma (HE×100); (**D**) Nevus (HE×100); (**E**) Cyst (HE×100); (**F**)Granuloma (HE×100).

**S-Figure 3. The common masses of the conjunctiva tumor. (A**) Lymphoma (HE×100); (**B**) Malignant epithelial (HE×200); (**C**) Squamous cell carcinoma (HE×100); (**D**)Nevus(HE×100); (**E**) Granuloma(HE×100); (**F**) Cyst (HE×100).


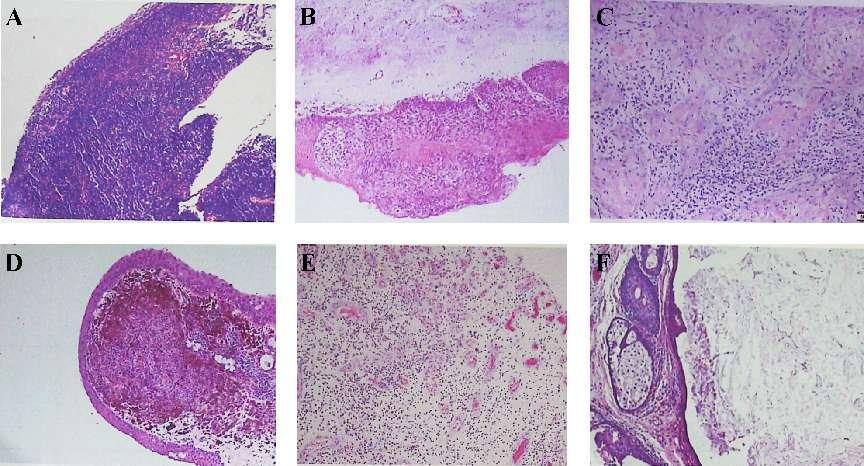


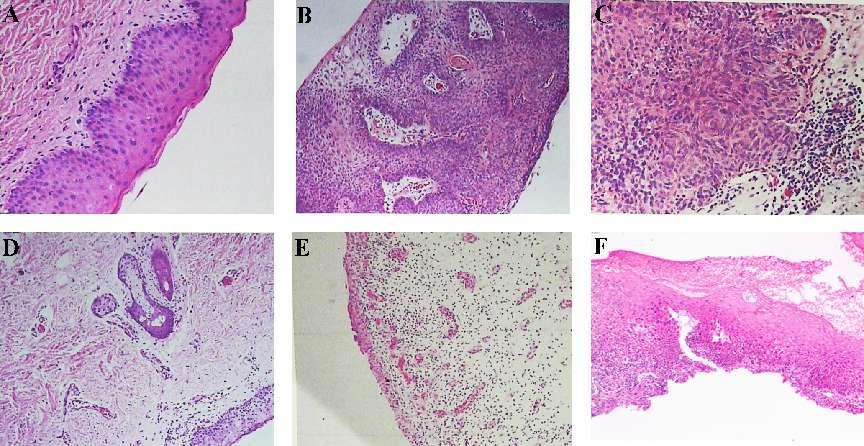
**S-Figure 4. The common masses of the corneal tumor**. (**A**) Malignant epithelium (HE×200); (**B**) Squamous cell carcinoma (HE×100);(**C**) Preinvasive carcinoma(HE×200); (**D**) Dermoid tumor (HE×100); (**E**) Granuloma(HE×100); (**F**) Hyperplasia(HE×100).


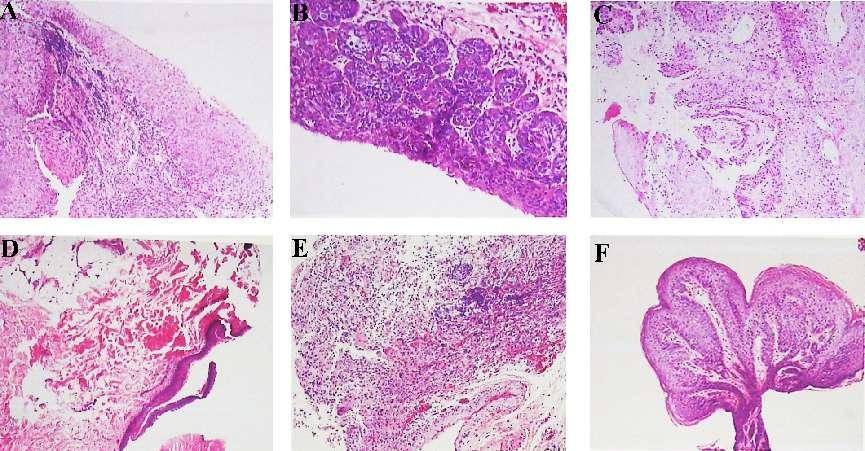
**S-Figure 5. The common masses of the corneal limbus tumor. (A**) Malignant epithelial (HE×100); (**B**) Epithelial carcinoma (HE×100); (**C**) Squamous cell carcinoma (HE×100); (**D**) Dermoid tumor (HE×100); (**E**) Granuloma (HE×100); (**F**) Squamous papilloma (HE×100).
